# Climate Change, Extreme Weather, and Intimate Partner Violence in East African Agrarian-Based Economies

**DOI:** 10.3390/ijerph20237124

**Published:** 2023-11-30

**Authors:** Leso Munala, Elizabeth M. Allen, Andrew J. Frederick, Anne Ngũnjiri

**Affiliations:** 1 Public Health Department, St. Catherine University, St. Paul, MN 55105, USA; ajfrederick231@stkate.edu; 2 LVCT Health, Nairobi P.O. Box 19835-00202, Kenya; anne.ngunjiri@lvcthealth.org

**Keywords:** gender-based violence, violence against women, flooding, drought, domestic violence, climate justice, Uganda, Mozambique, Zimbabwe, agriculture

## Abstract

Severe weather events can be a catalyst for intimate partner violence, particularly in agricultural settings. This research explores the association between weather and violence in parts of East Africa that rely on subsistence farming. We used IPUMS-DHS data from Uganda in 2006, Zimbabwe in 2010, and Mozambique in 2011 for intimate partner violence frequency and EM-DAT data to identify weather events by region in the year of and year prior to IPUMS-DHS data collection. This work is grounded in a conceptual framework that illustrates the mechanisms through which violence increases. We used logistic regression to estimate the odds of reporting violence in regions with severe weather events. The odds of reporting violence were 25% greater in regions with severe weather compared to regions without in Uganda (OR = 1.25, 95% CI: 1.11–1.41), 38% greater in Zimbabwe (OR = 1.38, 95% CI: 1.13–1.70), and 91% greater in Mozambique (OR = 1.91, 95% CI: 1.64–2.23). Our results add to the growing body of evidence showing that extreme weather can increase women’s and girls’ vulnerability to violence. Moreover, this analysis demonstrates that climate justice and intimate partner violence must be addressed together.

## 1. Introduction

Climate change is an urgent global issue with far-reaching consequences that disrupt various facets of life. It is well-established that climate change exacerbates environmental degradation and resource scarcity, resulting in significant socioeconomic disruptions [1]. Severe weather events are also a result of climate change, including acute droughts, water scarcity, severe fires, rising sea levels, flooding, melting polar ice, catastrophic storms, and dwindling biodiversity [2]. Hotter temperatures have caused extreme heat waves all around the world [3]. Longer dry seasons and droughts have reduced water availability [1,4], while prolonged rains have increased the frequency and intensity of floods [1,5]. The intensification of storms and typhoons has resulted from warmer oceans and rising sea levels [1,6].

Beyond the direct consequences of climate change related to environmental degradation, climate change has been linked to increased vulnerability towards food security, human health, poverty reduction, and economic growth [1]. This can potentially have far-reaching economic consequences, affecting industries such as tourism, fishing, and insurance [7,8,9]. Climate change and severe weather have the greatest impact on poor countries [10]. This is due to the fact that people in poor and middle-income countries usually rely heavily on natural resources for subsistence, such as agriculture or fishing, both of which are directly influenced by climate change [10,11,12]. Furthermore, low and middle-income countries’ limited access to the resources and infrastructure required to adapt to or mitigate the effects of climate change only serves to exacerbate the individual-level consequences that they must deal with [13].

Africa’s current contribution to global greenhouse gas emissions is negligible, but the continent is extremely susceptible to the effects of climate change [14,15]. Sub-Saharan Africa, for example, has experienced an increase in extreme climate conditions for the last few decades [15]. While eastern Africa is projected to face more extreme flooding events, western and southern Africa are likely to suffer from increased drought events [16,17,18]. African economies rely disproportionately on climate-vulnerable sectors such as agriculture, with 95 percent of African cropland being rainfed [19]. More specifically, East Africa has experienced rising temperatures, loss of water sources, and frequent heavy rains and droughts [20,21,22,23,24]. This directly impacts the majority of East Africans who rely on subsistence agriculture as a primary source of income, as diminishing water sources and rising temperatures make it increasingly impossible for them to sustain their livelihoods [24]. Flooding and droughts are some of the most severe and evident reactions to climate change, especially for the agricultural industry [25]. Climate change-related drought has led to over 90 million people facing food insecurity in East Africa. Wheat and corn are estimated to decrease yield by approximately 45 percent, and soybeans are projected to decrease yield by 20 percent by the year 2050 [24].

The impact of climate change is gendered-women, and girls are disproportionately affected by the loss of biodiversity, pollution, and natural disasters [26,27,28]. Gender inequality inherited from historical, socioeconomic developmental processes and entrenched social norms is a major factor exacerbating vulnerability to climate change impacts [29,30]. For example, in many parts of the world, women and children, typically girls, often perform daily household tasks such as gathering water and firewood. When droughts, deforestation, and floods decimate nearby supplies, women and children are forced to walk further to get these necessities, increasing their risk of sexual and physical violence [28,31]. As vulnerable populations struggle to maintain their food security and livelihoods, a reduction in the availability of vital natural resources can lead to an increase in transactional sex [32]. When crops are destroyed by droughts or floods, families in some countries have forced young females to marry early in exchange for food or livestock [33,34]. The aforementioned impact has significant mental health effects on women, exacerbating tension, anxiety, and emotional distress due to increased environmental hazards, displacement, and future uncertainty [35].

Climate change is a catalyst for violence around the world. Harsh climates contribute to social instability [36,37,38,39], and in some parts of the world, reports of intimate partner violence have increased after severe weather events [37,40]. The competition for diminishing resources, such as water and arable land, intensifies as global temperatures rise and weather patterns become more erratic [41]. This scarcity can fuel tensions and exacerbate existing social and political divides [39,41,42]. Extreme weather patterns can also put women and girls in more vulnerable positions and often lead to increases in violence against women and girls and intimate partner violence [43,44,45]. Following disasters, dwindling financial prospects and the threat of long-term unemployment cause stress and contribute to the rise in violence, particularly in agricultural settings [46,47]. Food insecurity due to meager harvests or the loss of livestock during severe weather events causes feelings of insecurity, anxiety, and stress among men who can no longer provide for their families [31,37,48,49]. Natural disasters like Hurricane Katrina in the United States and Cyclone Pam in Vanuatu have caused increased post-event violence. Similarly, extreme weather events such as heat waves, droughts, and flooding have also yielded more prevalent violence after these events [31,40,50,51,52].

**Figure 1:**
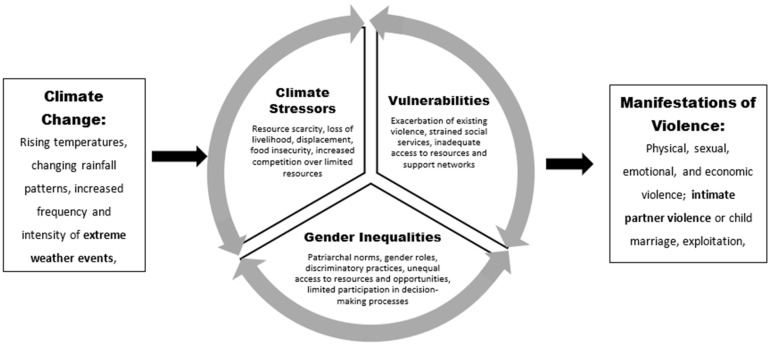
Conceptual Framework.

Based on previous research, we developed a conceptual framework for understanding the interlinkage between climate change, severe weather, and intimate partner violence, structured around three key components: (1) Climate Stressors, (2) Gender Inequalities and Power Dynamics, and (3) Amplification of Vulnerabilities. These components are interconnected and mutually reinforcing, shaping the experiences and vulnerabilities of women and girls in the context of climate change. The framework, shown in Figure 1, guides this research and the interconnectedness of climate change and violence.

The purpose of this analysis is to explore the relationship between extreme weather and intimate partner violence in agrarian-based economies. We focus on extreme weather events, specifically severe floods and droughts, rather than other climate stressors, as these events have the most acute impact on agriculture. Expanding upon previous research [21,22,40], this paper explores the correlation between severe weather and violence in larger portions of East Africa. Uganda, Zimbabwe, and Mozambique are three significantly agrarian countries, with 70 to 90 percent of the population engaged in agricultural activities, substantially higher than the global agricultural workforce of approximately 27 percent [53,54,55,56,57]. The majority of people in Uganda (69%), Zimbabwe (70%), and Mozambique (90%) depend on subsistence agriculture, with surplus food sold on the market for additional income [55,57,58,59,60]. Each of these three countries has a higher incidence of domestic violence than the global average of 33 percent [61,62]. These three countries’ agricultural landscapes make them optimal for investigating this association. This work adds to the growing body of evidence that climate change contributes to intimate partner violence in various agrarian settings.

## 2. Methods

We conducted an ecological study utilizing secondary data from the Integrated Public Use Microdata Series Demographic and Health Survey (IPUMS-DHS) and Emergency Events Database (EM-DAT) to conduct quantitative data analysis of severe weather events’ effects on intimate partner violence in Uganda, Zimbabwe, and Mozambique. IPUMS-DHS is a database that houses census and survey data from low and middle-income countries. IPUMS-DHS utilizes surveys collected at a household level in low and middle-income countries every three to five years. The surveys are collected from women ages 15–49 and have a series of demographic questions and public health questions, such as those related to intimate partner violence. The demographics and health-related data from IPUMS-DHS are downloadable in various data types [61]. EM-DAT is a weather database compiled from various sources, including UN agencies, non-governmental organizations, reinsurance companies, research institutes, and press agencies. EM-DAT stores information on more than 26,000 major disasters worldwide from 1900 to the present day [63].

IPUMS-DHS provided us with information on experiences of intimate partner violence. Our explanatory variables are reports of intimate partner violence measured as having experienced violence (physical, sexual, emotional, or any) in the past year. Additional dependent variables include whether the partner drinks alcohol, whether the partner works in agriculture and the partner’s level of education. We used EM-DAT to identify extreme weather events in Uganda, Zimbabwe, and Mozambique during the years of and the year prior to the DHS surveys. We defined severe weather events as our exposure variables, such as flooding lasting longer than five days, affecting more than 10,000 people, and droughts lasting one month or longer [61,63].

We used STATA statistical software version 17.0 BE-Basic Edition [64] to clean, merge, and manage data sets. We utilized the IPUMS-DHS surveys for experiences of intimate partner violence in Uganda in 2006, Zimbabwe in 2010, and Mozambique in 2011. The study population was limited to women ages 15–49 who had ever been married or lived with a man and who filled out the domestic violence survey questions. IPUMS-DHS collects data from different countries at different times. Thus, we did not have data from the same year for the three countries. We selected 2006, 2010, and 2011 for Uganda, Zimbabwe, and Mozambique, respectively, because they had the most robust weather data from EM-DAT (Uganda: one drought; Mozambique: three floods, one drought; Zimbabwe: one drought) and some of the most extensive IPUMS-DHS data related to intimate partner violence. We used the IPUMS-DHS variables for Uganda, Zimbabwe, and Mozambique to identify a region of residence and cross-referenced those variables with EM-DAT data to identify weather events in regions of IPUM-DHS data collection. We grouped variables related to intimate partner violence into four categories: physical, sexual, emotional, and total, which includes experiences of any form of intimate partner violence.

We calculated odds ratios and 95% confidence intervals using logistic regressions to determine the relationship between intimate partner violence and severe weather events. We conducted both crude and multivariate models controlling for whether the spouse worked in agriculture, whether the spouse drank alcohol, and the spouse’s education level.

## 3. Results

The 2006 Uganda DHS survey included 1739 women, the Zimbabwe 2010 survey had 5280 women, and the Mozambique DHS 2011 survey included 5824 women. The mean age for all three countries was approximately 26 years. The majority of respondents lived in rural areas, with Uganda having the greatest number of rural respondents (85%). Only in Uganda, a majority of respondents said that their partner consumed alcohol (56%), while a minority in Zimbabwe (47%) and Mozambique (39%) answered that their partner consumed alcohol. In all three countries, the majority of female respondents were married. On average, there was a higher level of educational attainment (secondary school or higher) in Zimbabwe for both men and women. In Uganda and Mozambique, the majority of women had completed primary school. Complete study demographics can be found in Table 1.

In Uganda, 61 percent of women reported experiences of any form of intimate partner violence in the previous year. In Zimbabwe and Mozambique, 37 percent and 43 percent of women reported some form of intimate partner violence, respectively. Complete study results can be found in Table 2 and Table 3 and summarized below.

The odds of respondents reporting any form of intimate partner violence were more likely among women who lived in regions that experienced severe weather events than those who did not live in regions that experienced severe weather events (Uganda OR = 1.23, 95% CI: 1.09–1.38; Zimbabwe OR = 1.28, 95% CI: 1.09–1.51; and Mozambique OR = 1.91, 95% CI: 1.64–2.23). Women whose partners drink alcohol also had increased odds of experiencing violence compared to those whose partners do not drink in all three countries (Uganda: OR = 2.19, 95% CI: 1.78–2.69; Zimbabwe OR = 1.81, 95% CI: 1.62–2.04; and Mozambique OR = 2.64, 95% CI: 2.35–2.96). However, the odds of reporting intimate partner violence were lowered by the partner’s education level only in Uganda (OR = 0.80, 95% CI: 0.67–0.96). The odds of reporting physical intimate partner violence were higher among severe weather in Zimbabwe (OR = 1.22, 95% CI: 1.00–1.48), which was not true for the other two countries. While the odds of emotional, intimate partner violence were higher among severe weather in Uganda and Mozambique (Uganda OR = 1.33, 95% CI: 1.19–1.50; Mozambique: OR = 2.17, 95% CI: 1.83–2.58), this was not true for Zimbabwe. Also, the odds of sexual intimate partner violence were greater among people who experienced severe weather in Uganda and Zimbabwe (Uganda OR = 1.36, 95% CI: 1.19–1.56; Zimbabwe: OR = 1.45, 95% CI: 1.13–1.85) though it was not true in Mozambique. Complete study results can be found in Table 2 and Table 3.

In all three countries, having a partner who drinks alcohol increased the odds of reported physical violence (Uganda OR = 2.87, 95% CI: 2.30–3.59; Zimbabwe OR = 2.00, 95% CI: 1.74–2.30; Mozambique OR = 2.58, 95% CI: 2.27–2.92) and emotional violence (Uganda OR = 2.07, 95% CI: 1.69–2.54; Zimbabwe: OR = 1.76, 95% CI: 1.55–2.01; Mozambique: OR = 2.22, 95% CI: 1.98–2.50). However, the odds reporting sexual intimate partner violence were greater for respondents whose partners drank in Zimbabwe and Mozambique (Zimbabwe OR = 1.49, 95% CI: 1.27–1.76; Mozambique: OR = 2.83, 95% CI: 2.25–3.55) while in Uganda it was not. Complete study results can be found in Table 2 and Table 3.

Ugandan respondents had lower odds of reporting any and every form of intimate partner violence if their partner had a higher level of education (Uganda Any form: OR = 0.80, 95% CI: 0.67–0.96; Physical: OR = 0.79, 95% CI: 0.0.66–0.95; Emotional: OR = 0.82, 95% CI: 0.69–0.98; Sexual: OR = 0.82, 95% CI: 0.67–0.99) compared to those whose partners had a lower level of education, which was not true for Zimbabwe and Mozambique. The odds of sexual intimate partner violence were lower if the respondent’s partner worked in agriculture in Mozambique (OR = 0.72, 95% CI: 0.55–0.93); this was not true in the other countries. Complete study results can be found in Table 2 and Table 3.

## 4. Discussion

This study adds to the growing body of evidence that severe weather and violence are linked [21,22,31,40,45,52,65,66]. Higher temperatures and extreme weather events, according to these studies, can increase the likelihood of intimate partner violence. Intimate partner violence occurs at a global rate of 33%, with Uganda (60.5%), Zimbabwe (36.6%), and Mozambique (43.2%) all having greater rates than the global norm [61,67]. In all three countries, extreme weather events were found to be linked with an increase in the reporting of intimate partner violence in Uganda (OR = 1.23, 95% CI: 1.09–1.38), in Zimbabwe (OR = 1.28, 95% CI: 1.09–1.51), and in Mozambique (OR = 1.91, 95% CI: 1.64–2.23). Interestingly, types of violence are not uniformly elevated across all three countries. This variation may be attributed to cultural norms and societal attitudes toward violence and gender roles; this may impact perceived definitions and, thus, reporting of types of violence. Across all three countries, working in agriculture was not associated with increased reports of intimate partner violence. This indicates that simply working in an agricultural setting alone does not necessarily drive violence. However, when severe weather events occur, reports of violence increase. This finding provides additional evidence that weather conditions serve as a catalyst for violence within agricultural communities rather than the inherent nature of agricultural activity.

Our data reveal significant correlations between alcohol consumption and the prevalence of intimate partner violence in Uganda, Zimbabwe, and Mozambique, corroborating previous research while revealing variations in the impact of these factors across countries [68,69,70,71]. In all three countries, if the respondent’s partner consumes alcohol, the respondent is statistically more likely to report intimate partner violence. Only in Uganda, the odds of intimate partner violence decreased with higher levels of education at a statistically significant level (OR = 0.72, 95% CI: 0.55–0.93). Though reports of violence in Zimbabwe or Mozambique were lower with higher levels of education, these results did not hold significance. This was surprising given that other studies have shown that education is associated with lower rates of intimate partner violence [68,69,71,72,73,74]. These results are important to the understanding of the intersections between climate and violence but also contribute to the broader context of climate change.

Severe weather events and their consequences shed light on pre-existing socioeconomic vulnerabilities [75]. The available literature indicates that climate change has contributed to the widening of existing health inequalities between and within populations [48,67]. As our results shed light on the connection between severe weather events and intimate partner violence, there are many mechanisms that increase vulnerability and through which violence can be heightened. For example, climate change-induced displacement and forced migration are one mechanism for widening inequalities. As global temperatures rise and weather patterns become erratic, the competition for dwindling resources, such as water and arable land, intensifies [40]. Consequently, people are forced to relocate to regions that offer greater availability of resources. 

Moreover, the limited availability of these important resources might give rise to disputes, intensifying tensions and worsening pre-existing social and political rifts [39,41,42]. We also see a multifaceted impact on food security. One notable consequence is a reduction in food production due to diminished agricultural yields and crop devastation. Reduced agricultural output impacts the market value of food goods, which can make costs out of reach for some people [23,24,25,76]. This can result in engaging in transactional sexual relationships [32] or early marriage among females as a means to secure sustenance and financial resources [33,34]. Reduced outputs also have devastating economic effects on the lives of families. In countries such as Uganda, Zimbabwe, and Mozambique, where a significant portion of the population is engaged in agricultural activities, individuals who work as subsistence farmers or sell surplus crops at local markets face severe financial repercussions in the event of crop failure. These consequences include the loss of income derived from selling crops at markets and the potential for unemployment in agriculture [9,77]. We have seen from previous literature feelings of insecurity, anxiety, and stress among men who can no longer provide for their families can increase violence at home [31,37,48,49].

Our results corroborate previous research that shows that extreme weather patterns can put women and girls in more vulnerable positions and often lead to increases in violence [45]. The impacts of climate change worsen pre-existing social inequalities, specifically for women and girls. When livelihood depends on agriculture and natural resources, women and girls are more vulnerable because of gender inequities and unfair social norms related to climate change [30,78,79,80]. In many parts of the world, it is the woman’s responsibility to obtain living necessities such as water, food, and fuel. When children are responsible for performing these tasks and their daily chores consume substantial amounts of time and energy, they miss school, and school-age girls are more likely to drop out of school to help the family with household responsibilities than their brothers [81,82].

Numerous policies and initiatives to resolve environmental concerns often disregard or even cause harm to women and other marginalized communities [78,83]. Even though they are among those most susceptible to its outcomes, women are often left out of the decision-making and thus unable to effect changes to improve their health, safety, and survival [30,31,37,80]. The results of this analysis further support the need to not only articulate the impact of climate change on women but also elevate the experiences and voices of women who are impacted. Policymakers must include women in decision-making and collaborate with policymakers in the global south [84]. It is imperative that policymakers utilize weather and intimate partner violence research to inform their decision-making with empirical evidence. One key approach involves empowering women and girls by enhancing their access to education, healthcare, and economic opportunities. By increasing their agency and resilience, women and girls can better navigate the impacts of climate change and reduce their vulnerability to violence. Additionally, strengthening legal frameworks and institutions to ensure gender-responsive policies and laws is essential. Community-based initiatives that engage women and girls in decision-making processes, such as climate change planning and disaster risk reduction, are also vital.

Our conceptual framework posits that stressors, gender norms, and existing vulnerabilities are mechanisms that increase violence during and after extreme weather events. The existence of vulnerable populations, as evidenced by the high prevalence of violence, is compounded by additional stressors in these agrarian societies that are heavily dependent on agriculture. Furthermore, these countries enforce gender norms that assign women and girls the responsibility of gathering natural resources. Previous researchers have noted many pathways that contribute to the escalation of violence, which are consistent with our conceptual framework.

There are limitations to our study that should be considered when interpreting the results of this analysis. EM-DAT does not include GPS coordinates for every weather event. We could only identify extreme weather events by region, which could potentially lead to misclassification of weather events. Nevertheless, any potential misclassification would not differ significantly between women who reported intimate partner violence and those who did not. As a result, it would not have a considerable impact on the measure of association. We also used weather data that affected a large number of people to help mitigate potential exposure misclassification. Notwithstanding these constraints, we maintain a sense of assurance regarding our findings, as our data align with the documented connections established between covariates and reports of violence.

This work lays the foundation for future research in which it is important to understand the intersection between climate change and violence. The next steps should elevate the lived experiences of women whose livelihood depends on agriculture and who experience extreme weather events. Not only would this provide primary data on the mechanisms between weather and violence, but it also moves from an ecological to an individual-level approach. Individual-level research would further validate our conceptual framework. Future work should also contextualize these results in existing IPV policies and offer policy solutions.

## 5. Conclusions

We highlighted a significant association between severe weather events and reports of intimate partner violence in three East African agrarian-based economies. This work underscores the significance of addressing fundamental issues, such as economic strain and displacement resulting from extreme weather occurrences, in order to efficiently mitigate intimate partner violence within agricultural environments. Addressing the vulnerability of climate change is crucial to ensuring the well-being and safety of communities, particularly for marginalized groups such as women and girls. Climate change intensifies existing social, economic, and gender inequalities, making these vulnerable populations even more susceptible to its adverse effects. These findings highlight the importance of addressing violence against women as a central component of the movement for climate justice. Therefore, we cannot ignore the voices of women and the impact of climate change on their quality of life. If we disregard the relationship between climate change and violence, we will not make any progress in the fight for climate justice.

## Figures and Tables

**Table 1:** Demographics of the Study Population.

	Uganda (*n* = 1739)	Zimbabwe (*n* = 5280)	Mozambique (*n* = 5824)
Characteristics	Survey Year 2006 N (%)	Survey Year 2010 N (%)	Survey Year 2011 N (%)
Urban/Rural			
Urban	249 (14.3)	1666 (31.6)	1978 (34.0)
Rural	1490 (85.7)	3614 (68.4)	3846 (66.0)
Age			
Mean age	26.5	26.5	26.4
15–19	109 (6.3)	317 (6.0)	589 (10.1)
20–29	749 (43.1)	2299 (43.5)	2287 (39.3)
30–39	580 (33.4)	1753 (33.2)	1922 (33.0)
40–49	301 (17.3)	911 (17.3)	1026 (17.6)
Marital Status			
Married	1137 (65.4)	4203 (79.6)	3048 (52.3)
Living Together	338 (19.4)	208 (3.9)	1832 (31.5)
Other	264 (15.2)	869 (16.5)	944 (16.2)
Woman’s Education Level			
None	432 (24.9)	161 (3.1)	1950 (33.5)
Primary	1049 (60.3)	1807 (34.2)	3040 (52.2)
Secondary or Higher	258 (14.8)	3312 (62.7)	834 (14.3)
Partner’s Education Level			
None	193 (11.1)	152 (2.9)	1219 (20.9)
Primary	997 (57.3)	1191 (22.5)	2904 (49.9)
Secondary or Higher	489 (28.1)	3811 (72.2)	1284 (22.0)
Missing	60 (3.5)	126 (2.4)	417 (7.2)
Woman’s Occupation			
Unemployed	137 (7.9)	2939 (55.7)	2854 (49.0)
Professional, Technical, Managerial	47 (2.7)	166 (3.1)	161 (2.8)
Agricultural	1208 (69.7)	568 (10.7)	1830 (31.4)
Manual Labor	95 (5.5)	468 (8.9)	66 (1.1)
Other	246 (14.2)	1118 (21.2)	894 (15.4)
Missing	3 (0.3)	21 (0.4)	19 (0.3)
Partner’s Occupation			
Unemployed	N/A	793 (15.0)	213 (3.7)
Professional, Technical, Managerial	126 (7.2)	378 (7.2)	439 (7.5)
Agricultural	984 (56.6)	915 (17.3)	2368 (40.7)
Manual Labor	316 (18.2)	2094 (39.7)	1491 (25.6)
Other	290 (16.7)	1048 (19.8)	1202 (20.6)
Missing	23 (1.3)	52 (1.0)	111 (1.9)
Partner drinks alcohol			
No	752 (43.2)	2765 (52.4)	3526 (60.5)
Yes	985 (56.7)	2515 (47.6)	2298 (39.5)
Missing	2 (0.1)	N/A	N/A
* Time to water and back (minutes)			
0–15 and on premises	1075 (62.0)	3546 (67.2)	2805 (48.2)
16–30	409 (23.5)	1030 (19.5)	1430 (24.5)
31–45	56 (3.2)	201 (3.8)	517 (8.9)
46–60	110 (6.3)	289 (5.5)	517 (8.9)
61+	25 (1.4)	166 (3.1)	497 (8.5)
Do Not Know	4 (0.2)	48 (0.9)	58 (1.0)
Missing	60 (3.4)	N/A	N/A
Domestic Violence			
Yes	1052 (60.5)	1933 (36.6)	2517 (43.2)

* Time to water and back indicates the amount of time (in minutes) that women report traveling to and from their residence to collect water.

**Table 2:** Uganda, Zimbabwe, and Mozambique Odds Ratio Intimate Partner Violence by Severe Weather Events.

	Uganda	Zimbabwe	Mozambique
	Any Forms IPV	Any Forms IPV	Any Forms IPV
	OR (95%CI)	OR (95%CI)	OR (95%CI)
Unadjusted			
Severe Weather	1.17 (1.04–1.30)	1.27 (1.09–1.47)	2.13 (1.84–2.45)
Partner works in agriculture	1.32 (1.08–1.60)	1.00 (1.00–1.01)	1.00 (1.00–1.01)
Works in agriculture	1.3 (1.06–1.60)	1.31 (1.09–1.56)	1.07 (0.95–1.19)
Partner drinks	2.17 (1.78–2.64)	1.83 (1.31–2.05)	2.69 (2.41–3.00)
Urban/rural	1.57 (1.20–2.06)	1.07 (0.95–1.21)	0.76 (0.68–0.84)
Level of education	0.84 (0.72–0.98)	0.88 (0.80–0.98)	1.14 (1.05–1.23)
Partner’s level of education	0.73 (0.62–0.86)	1.00 (0.89–1.11)	1.12 (1.03–1.21)
Adjusted			
* Severe Weather	1.23 (1.09–1.38)	1.28 (1.09–1.51)	1.91 (1.64–2.23)
Partner works in agriculture	1.16 (0.93–1.44)	1.14 (0.98–1.32)	0.89 (0.79–1.02)
Partner drinks	2.19 (1.78–2.69)	1.81 (1.62–2.04)	2.64 (2.35–2.96)
Partner’s level of education	0.8 (0.67–0.96)	1.07 (0.95–1.20)	1.00 (0.91–1.10)

* Severe weather is defined as any flood lasting more than five days and affecting more than 10,000 people or any drought lasting one month or longer.

**Table 3:** Uganda, Zimbabwe, and Mozambique Odds Ratio of Experiencing Types of Intimate Partner Violence.

	Uganda			Zimbabwe			Mozambique		
	Physical Violence	Emotional Violence	Sexual Violence	Physical Violence	Emotional Violence	Sexual Violence	Physical Violence	Emotional Violence	Sexual Violence
	OR (95%CI)	OR (95%CI)	OR (95%CI)	OR (95%CI)	OR (95%CI)	OR (95%CI)	OR (95%CI)	OR (95%CI)	OR (95%CI)
Unadjusted									
Severe Weather	0.98 (0.88–1.10)	1.28 (1.14–1.43)	1.30 (1.14–1.48)	1.21 (1.01–1.45)	1.13 (0.95–1.34)	1.43 (1.13–1.81)	1.31 (1.12–1.54)	2.39 (2.03–2.80)	1.39 (1.04–1.86)
Partner works in agriculture	1.40 (1.14–1.71)	1.15 (0.95–1.40)	1.27 (1.02–1.59)	1.00 (1.00–1.01)	1.00 (1.00–1.01)	1.00 (0.99–1.00)	1.00 (1.00–1.01)	1.00 (1.00–1.01)	0.99 (0.98–1.00)
Works in agriculture	1.36 (1.09–1.70)	1.12 (0.91–1.38)	1.34 (1.05–1.70)	1.15 (0.93–1.41)	1.16 (0.95–1.41)	1.24 (0.97–1.59)	0.92 (0.81–1.04)	0.99 (0.88–1.12)	0.55 (0.42–0.70)
Partner drinks	2.96 (2.39–3.67)	2.02 (1.66–2.45)	1.25 1.00–1.56	2.02 (1.76–2.31)	1.76 (1.55–2.00)	1.47 (1.25–1.73)	2.46 (2.18–2.78)	2.32 (2.08–2.60)	2.6 (2.10–3.22)
Urban/rural	1.71 (1.25–2.32)	1.18 (0.90–1.54)	1.51 (1.08–2.10)	1.04 (0.90–1.20)	1.00 (0.87–1.14)	1.18 (0.98–1.41)	0.81 (0.72–0.92)	0.74 (0.66–0.83)	0.91 (0.73–1.13)
Level of education	0.67 (0.57–0.79)	0.92 (0.79–1.07)	0.88 (0.74–1.05)	0.89 (0.79–1.00)	0.91 (0.81–1.02)	0.93 (0.80–1.07)	1.06 (0.97–1.16)	1.15 1.06–1.25)	0.96 (0.82–1.12)
Partner’s level of education	0.69 (0.58–0.82)	0.77 (0.66–0.91)	0.78 (0.65–0.93)	0.99 (0.87–1.13)	0.94 (0.83–1.07)	1.05 (0.90–1.24)	0.98 (0.90–1.08)	1.14 (1.05–1.24)	1.05 (0.90–1.23)
Adjusted									
Severe Weather	1.05 (0.93–1.19)	1.33 (1.19–1.50)	1.36 (1.19–1.56)	1.22 (1.00–1.48)	1.17 (0.98–1.41)	1.45 (1.13–1.85)	1.15 (0.97–1.37)	2.17 (1.83–2.58)	1.03 (0.75–1.41)
Partner works in agriculture	1.17 (0.92–1.47)	1.03 (0.83–1.29)	1.18 (0.93–1.51)	1.05 (0.88–1.25)	0.97 (0.82–1.15)	1.17 (0.95–1.45)	0.91 (0.78–1.05)	0.91 (0.80–1.04)	0.72 (0.55–0.93)
Partner drinks	2.87 (2.30–3.59)	2.07 (1.69–2.54)	1.24 (0.99–1.56)	2.00 (1.74–2.30)	1.76 (1.55–2.01)	1.49 (1.27–1.76)	2.58 (2.27–2.92)	2.22 (1.98–2.50)	2.83 (2.25–3.55)
Partner’s level of education	0.79 (0.66–0.95)	0.82 (0.69–0.98)	0.82 (0.67–0.99)	1.06 (0.92–1.21)	0.99 (0.87–1.12)	1.15 (0.97–1.36)	0.91 (0.82–1.00)	1.04 (0.95–1.14)	0.90 (0.76–1.07)

## Data Availability

The data that support the findings of this study are openly available in IPUMS DHS: https://www.idhsdata.org/idhs/ and EM-DAT: https://www.emdat.be/ (accessed on 15 June 2023). These data were derived from the following resources available in the public domain: weather data for Uganda, Zimbabwe, and Mozambique https://public.emdat.be/data (accessed on 15 June 2023) and DHS survey data for Uganda, Zimbabwe, and Mozambique https://www.idhsdata.org/idhs-action/extract_requests/summary (accessed on 15 June 2023).
